# Prognostic impact of gross tumor volume during radical radiochemotherapy of locally advanced non-small cell lung cancer—results from the NCT03055715 multicenter cohort study of the Young DEGRO Trial Group

**DOI:** 10.1007/s00066-020-01727-4

**Published:** 2021-01-07

**Authors:** C. Ostheimer, M. Mäurer, N. Ebert, D. Schmitt, D. Krug, R. Baumann, C. Henkenberens, F. A. Giordano, L. Sautter, Guerra López, D. F. Fleischmann, M. Niyazi, L. Käsmann, D. Kaul, A. H. Thieme, C. Billiet, S. Dobiasch, C. R. Arnold, M. Oertel, J. Haussmann, T. Gauer, Y. Goy, C. Suess, S. Ziegler, C. M. Panje, C. Baues, M. Trommer, T. Skripcak, D. Medenwald

**Affiliations:** 1 Department of Radiation Oncology, Faculty of Medicine, Martin Luther University Halle-Wittenberg, Ernst-Grube-Straße 40, 06110 Halle (Saale), Germany; 2 Department of Radiation Oncology, University Medical Center Jena, Jena, Germany; 3 Department of Radiation Oncology, University Medical Center Dresden, Dresden, Germany; 4 OncoRay—National Center for Radiation Research in Oncology, Dresden, Germany; 5 Department of Radiation Oncology, University Hospital Heidelberg, Heidelberg, Germany; 6 National Center for Radiation Research in Oncology (NCRO), Heidelberg, Germany; 7 Heidelberg Institute for Radiation Oncology (HIRO), Heidelberg, Germany; 8 Department of Radiation Oncology, University Medical Center Schleswig-Holstein, Kiel, Germany; 9 Department of Radiation and Special Oncology, Hannover Medical School, Hannover, Germany; 10 Department of Radiation Oncology, University Medical Center Mannheim, Mannheim, Germany; 11 Department of Radiation Oncology, Hospital Universitario Virgen del Rocío, Sevilla, Spain; 12 Department of Radiation Oncology, LMU Munich, Munich, Germany; 13 partner site Munich, German Cancer Consortium (DKTK), Munich, Germany; 14 German Cancer Research Center (DKFZ), Heidelberg, Germany; 15 Department of Radiation Oncology, University of Lübeck, Lübeck, Germany; 16 Department of Radiation Oncology, Charité School of Medicine, Berlin, Germany; 17 Campus Virchow-Klinikum, University Hospital, Berlin, Germany; 18 Department of Radiation Oncology, Iridium Kankernetwerk, Antwerp, Belgium; 19 Department of Radiation Oncology, Technische Universität München, Munich, Germany; 20 Department of Therapeutic Radiology and Oncology, Medical University of Innsbruck, Innsbruck, Austria; 21 Department of Radiation Oncology, University Medical Center Muenster, Muenster, Germany; 22 Department of Radiation Oncology, University Medical Center Düsseldorf, Dusseldorf, Germany; 23 Department of Radiotherapy and Radio-Oncology, University Medical Center Hamburg-Eppendorf, Hamburg, Germany; 24 Department of Radiation Oncology, University Medical Center Regensburg, Regensburg, Germany; 25 Department of Radiation Oncology, University Medical Center Erlangen, Erlangen, Germany; 26 Department of Radiation Oncology, Kantonsspital St. Gallen, St. Gallen, Switzerland; 27 Department of Radiation Oncology and Cyberknife Center, University of Cologne, Cologne, Germany; 28 German Cancer Consortium (DKTK), Dresden, Germany

**Keywords:** Non-small-cell lung cancer, Radiochemotheraoy, Gross tumor volume, Prediction, Overal survival

## Abstract

**Background:**

In radical radiochemotherapy (RCT) of inoperable non-small-cell lung cancer (NSCLC) typical prognostic factors include T- and N-stage, while there are still conflicting data on the prognostic relevance of gross tumor volume (GTV) and particularly its changes during RCT. The NCT03055715 study of the Young DEGRO working group of the German Society of Radiation Oncology (DEGRO) evaluated the prognostic impact of GTV and its changes during RCT.

**Methods:**

A total of 21 university centers for radiation oncology from five different European countries (Germany, Switzerland, Spain, Belgium, and Austria) participated in the study which evaluated *n* = 347 patients with confirmed (biopsy) inoperable NSCLC in UICC stage III A/B who received radical curative-intent RCT between 2010 and 2013. Patient and disease data were collected anonymously via electronic case report forms and entered into the multi-institutional RadPlanBio platform for central data analysis. GTV before RCT (initial planning CT, GTV1) and at 40–50 Gy (re-planning CT for radiation boost, GTV2) was delineated. Absolute GTV before/during RCT and relative GTV changes were correlated with overall survival as the primary endpoint. Hazard ratios (HR) of survival analysis were estimated by means of adjusted Cox regression models.

**Results:**

GTV1 was found to have a mean of 154.4 ml (95%CI: 1.5–877) and GTV2 of 106.2 ml (95% CI: 0.5–589.5), resulting in an estimated reduction of 48.2 ml (*p* < 0.001). Median overall survival (OS) was 18.8 months with a median of 22.1, 20.9, and 12.6 months for patients with high, intermediate, and low GTV before RT. Considering all patients, in one survival model of overall mortality, GTV2 (2.75 (1.12–6.75, *p* = 0.03) was found to be a stronger survival predictor than GTV1 (1.34 (0.9–2, *p* > 0.05). In patients with available data on both GTV1 and GTV2, absolute GTV1 before RT was not significantly associated with survival (HR 0–69, 0.32–1.49, *p* > 0.05) but GTV2 significantly predicted OS in a model adjusted for age, T stage, and chemotherapy, with an HR of 3.7 (1.01–13.53, *p* = 0.04) per 300 ml. The absolute decrease from GTV1 to GTV2 was correlated to survival, where every decrease by 50 ml reduced the HR by 0.8 (CI 0.64–0.99, *p* = 0.04). There was no evidence for a survival effect of the relative change between GTV1 and GTV2.

**Conclusion:**

Our results indicate that independently of T stage, the re-planning GTV during RCT is a significant and superior survival predictor compared to baseline GTV before RT. Patients with a high absolute (rather than relative) change in GTV during RT show a superior survival outcome after RCT.

**Supplementary Information:**

The online version of this article (10.1007/s00066-020-01727-4) contains supplementary material, which is available to authorized users.

## Introduction

Locally advanced non-small-cell lung cancer (NSCLC), i.e., Union for International Cancer Control (UICC) stage III, accounts for about 30% of all lung cancer cases and comprises a highly heterogeneous patient entity. This complicates finding the optimal treatment approach for the individual patient with regards to the different available modalities. Surgical resection for operable cases and definitive concurrent chemoradiotherapy (CRT) for inoperable patients remain the cornerstones in the interdisciplinary treatment of stage III NSCLC. Trials evaluating a bimodality approach (surgery followed by consolidation chemotherapy) failed to prove superiority over CRT, which offers 5‑year survival rates of 16–40% with clinically acceptable treatment toxicity [1]. However, treatment outcomes after definitive CRT are still suboptimal, with a significant number of patients who will eventually develop local recurrence. Thus, prognostic and predictive factors before and during RT are strongly needed to select the best therapeutic approach in order to increase the patients’ probability of survival.

The prognostic relevance of the gross tumor volume (GTV) in radiotherapy (RT) of locally advanced (stage III) non-small-cell lung cancer (NSCLC) is controversially discussed in the literature. Although an increasing tumor volume correlates with a higher T stage [2] in the TNM classification, there is no direct correlation. Preliminary evidence suggests the GTV to be an important outcome factor, albeit not dominant over the T stage [3], which may be of importance when surgical therapy is not the first choice, as the TNM classification is primarily obtained from surgical centers. Several studies investigated the prognostic impact of baseline GTV detected before RT on outcome and survival [4]. Available evidence suggests that especially the GTV at the beginning of therapy acts as a statistically significant prognostic indicator regarding overall survival (OS) and/or local tumor control [2, 4–13].

A direct comparison between different studies is, however, hampered due to the size of the available datasets and large variations in measurement timepoints during therapy as well as the employed definition of the tumor volume.

While the majority of the studies suggests a prognostic association of pre-treatment GTV with outcome after RT of advanced NSCLC, larger studies focusing on more homogeneous patient cohorts are necessary to determine the prognostic and predictive quality of pre-RT (baseline) GTV [4].

The primary objective of our multicentric study is to validate the predictive role of tumor volume change under RT in stage III NSCLC undergoing definitive chemoradiation. Secondary objectives include the estimation of the effect of tumor volume before and during chemoradiotherapy on overall survival.

## Methods

### Study population, treatment, and participating institutions

This retrospective observational cohort study (NCT03055715) was conducted by the Young DEGRO Trial Group (yDEGRO) of the German Society for Radiation Oncology (DEGRO). Twenty-one university centers for radiation oncology in Germany (*n* = 17), Spain (*n* = 1), Switzerland (*n* = 1), Belgium (*n* = 1) and Austria (*n* = 1) participated in the study. A total of *n* = 347 patients who were consecutively treated at all institutions with curative-intent radiation therapy (with/without chemotherapy) during the accrual period (January 1, 2010 to December 31, 2013) were analyzed.

Inclusion criteria were (1) inoperable UICC stage III A or B NSCLC (AC or SCC) confirmed by biopsy, (2) CT-based 3D radiation treatment planning (PET- or PET-CT-based if available), (3) completed curative-intent radiotherapy ± chemotherapy (planned total dose ≥60 Gy normofractionated or ≥50 Gy hypofractionated), and (4) age ≥18 years. Patients with a secondary malignancy within 5 years prior to the diagnosis of NSCLC, patients who received stereotactic body radiotherapy, patients who did not complete the full course of treatment, and patients who received neoadjuvant CT (i.e., before RT) were excluded from the study.

The local ethics (reference number: 2017-15) and data protection committees of the participating institutions approved the study protocol and gave their positive vote for the study, which was carried out in accordance with the declaration of Helsinki of 1975 (as revised in 2008).

Patient, treatment, and clinical data were extracted from the patients’ clinical records at the participating sites and collected using electronic case report forms (eCRF) which were stored in the RadPlanBio database of the German Cancer Consortium (DKTK) and the German Cancer Research Center (DKFZ) [14]. An intensive validation process was performed to check for implausible and incorrect values. This process was based on statistical approaches and spot checks. Written informed consent of all patients was available prior to data acquisition and analysis.

Staging was based on the TNM classification of malignant tumors (7th edition 2010).

### Detection and definition of gross tumor volume and toxicity

GTV included the gross tumor volume (without lymph nodes) as detectable in intravenous (iv) contrast-enhanced CT/PET-CT and was reported in millimeters. Where PET was available, PET-CT co-registration was hardware (integrated PET-CT scanner) or software based (with the need for repositioning of the patient), according to the equipment situation of the institutions. The basal GTV1 was delineated in the planning CT, which was obtained before the start of RT and correlated with PET, if existing. “Adaptive RT” (i.e., reducing the target volume during RT) was not practiced in the patient cohort analyzed in this study but in patients where a re-planning CT was available from radiation boost planning (after the patients received 40–50 Gy of their total planned dose), GTV2 was obtained from this re-planning CT.

Relative and absolute GTV changes refer to the relative or absolute GTV increase or decrease of GTV from GTV2 in relation to basal GTV1. In survival plots, values were defined as low, medium, and high according to the 25 and 75% quantile, respectively.

### Statistics

To assess the prognostic value of GTV1 and GTV2, frailty survival methods with study center as the shared frailty were used. Thus, it was possible to account for heterogeneity in GTV contouring and to consider covariates within the model in relation to study site. Such a model accounts for the fact that the delineation of the gross tumor volume might differ systematically between but less within institutions. Thus, the random variation between institutions is added as a further level. This is an extension of conventional models, which only consider random variation between subjects.

In the models assessing the prognostic value of GTV2, it was adjusted for the pre-treatment GTV (GTV1), allowing for evaluation of the effect of GTV2 independently from GTV1, which is thus kept constant across the subject in the statistical analyses.

Models were additionally adjusted for T stage (N stage was not included in the model as the gross volume of the primary tumor volume is unaffected by involvement of lymphatic nodes), chemotherapy, age, RT dose (by treatment given), histology (AC or SCC), grading, and pulmonary comorbidities. Additionally, a sensitivity analysis which was adjusted for pre-treatment PET-CT was performed.

In the Kaplan–Meier plots, the 25, 50, and 75% quantiles were used to define the patient groups with low, intermediate, and high GTV values, and strong, intermediate, and weak GTV decrease from GTV1 before RT and GTV2 during RT, respectively. Hazard ratios (HR) for survival comparisons between patient groups are reported with a 95% confidence interval (CI).

A sensitivity analysis was performed, which considered only patients with conventional fractionation (single dose of 2 or 1.8 Gy).

To identify non-linear relations in the statistical models because of skewed data, the martingale residuals were plotted (see Supplementary Information) and no evidence for a deviation from the assumption of linearity was found.

In our analyses, it was refrained from adjusting for multiple tests due to the following reasons: The only primary objective of our study was to estimate the predictive value of tumor volume change in terms of OS. All other analyses are secondary. Furthermore, several analyses that were all concerned with the primary objective but encompass different analytical methods were performed. Because of correlated analyses, established correction methods cannot be applied.

We additionally performed a sensitivity analysis in order to respect the lymph node status by adjusting for N status rather than T status in the respective regression models. All analyses were performed using SAS version 9.4 (Cary, NC, USA).

## Results

### Sociodemographic patient data, treatment, and clinical patient characteristics

Overall, 347 patients with locally advanced NSCLC were analyzed. Of these, 294 (84.7%) were treated in Germany, 22 (6.3%) in Belgium, 17 (4.9%) in Spain, 11 (3.2%) in Austria, and 3 (0.9%) in Switzerland. General patient and disease characteristics are summarized in Table 1.

**Table 1:** Sociodemographic patient and disease characteristics

	Patient number (%)
*Sex*	
Male	273 (78.7)
Female	74 (21.3)
*Age* (years), mean (SD)	67.2 (10.7)
*Pack years*, mean (SD)	38.2 (25.1)
*UICC stage*	
IIIA	174 (50.1)
IIIB	173 (49.9)
*Histology*	
AC	136 (39.3)
SCC	195 (56.4)
NS	–
*Grading*^a^	
1	7 (2)
2	104 (30)
3	120 (34.6)
4	5 (1.4)
NA	111 (32)
*T stage*	
T1	32 (9.3)
T2	63 (18.2)
T3	106 (30.6)
T4	144 (41.6)
Tx	2 (0.6)
*N stage*	
N0	32 (9.3)
N1	42 (12.1)
N2	172 (49.7)
N3	97 (28)
Nx	3 (0.9)

*AC* adenocarcinoma, *SCC* squamous cell carcinoma, *NS* non-small-cell lung cancer, not further specified, *NA* not available/missing

^a^Well, moderate, poor, undifferentiated (1–4)

Mean age of the patient cohort ranged from 40.5–91 years with 25, 50, and 75% percentiles of packyears of 20, 40, and 50, respectively.

The mean total radiation dose was 63.6 (range 45–75) Gy, with 301 patients (87%) having received a total dose of 60 Gy or higher, while 45 patients (13%) were treated with a cumulative dose of less than 60 Gy, of whom 20 received hypofractionated radiotherapy. 3 patients (0.8%) received a total dose of 45 or 46 Gy, less than the intended 50 Gy. The 25, 50, and 75% percentiles for the total dose were 60, 66, and 66 Gy. Median dose per fraction was 2 Gy (range 1.2–7.5). Mean fraction number was 31 (range 8–49, SD 5.6).

RT was combined with concurrent CHT in 250 patients (72.2%), 96 patients (27.8%) received sequential CRT. 75 patients (30%) received combined cisplatin-vinorelbine CHT, 48 (19.2%) carboplatin-vinorelbine, 52 (20.8%) carboplatin-docetaxel, and 75 (30%) other chemotherapy doublet combinations.

In 314 (90.8%) patients conventional fractionation was used, 7 (2%) patients were treated with hyperfractionated regimens, and 5 patients (1.5%) received a simultaneous-integrated boost (SIB) concept. 20 patients (5.7%) received other RT concepts.

### Gross tumor volume, its intra-therapeutic change, and association with overall survival

Median overall survival (OS) was 18.8 months for the total cohort, mean follow-up time in living patients was 22 (SD 19.5) months, with 239 patients (68.1%) already deceased, 51 (14.7%) alive, and 54 (15.6%) lost during follow-up.

Mean GTV1 before RT was 154.4 (range 1.5–877, SD 160.7) ml with the 25, 50, and 75% quantiles being 47.1, 47.8, and 220 ml, respectively. Mean GTV2 before radiation boost initiation was 106.2 (0.5–589.5, SD 105) ml with 25, 50, and 75% quantiles of 35.7, 35.72 and 133 ml, respectively. The difference between GTV1 and GTV2 was statistically significant (*p* < 0.001, considering only those patients where GTV1 and GTV2 were both recorded).

**Fig. 1:**
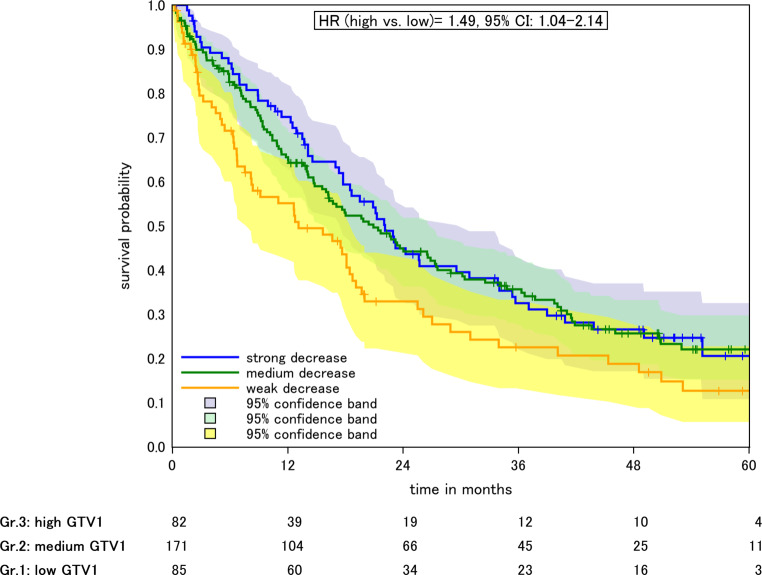
Kaplan–Meier plot of OS according to absolute GTV1 before radiotherapy. Low, medium, and high GTV1 referring to the 25 and 75% quantiles. *Colored areas* 95% confidence intervals (*CI*)

Median OS was 22.1, 20.9, and 12.6 months in patients with low, intermediate, and high baseline GTV1 before RT, Fig. 1. The hazard ratio (HR) for death for patients with intermediate (47.8–217.6 ml) and high GTV1 (220–877 ml) vs. low GTV1 (<47.1 ml) was 1.04 (95% CI 0.76–1.42, *p* > 0.05) and 1.49 (95% CI 1.04–2.14, *p* = 0.03), respectively. In the model adjusted for T‑stage, chemotherapy, age, RT dose, histology, and grading, the HR for death was 1.02 (0.71–1.44, *p* > 0.05) for low vs. intermediate GTV1 and 1.34 (0.9–2, *p* > 0.05) for low vs. high GTV1.

**Fig. 2:**
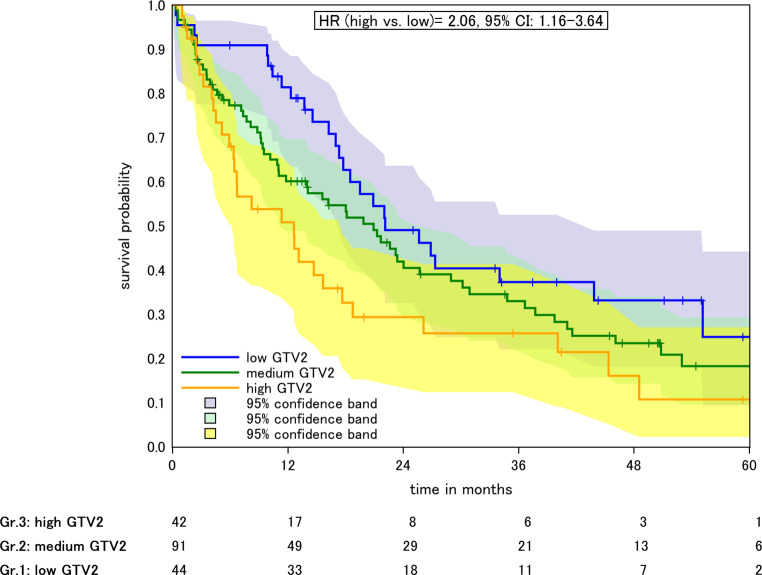
Kaplan–Meier plot of OS according to GTV2 before boost radiotherapy. Low, medium, and high GTV2 referring to the 25 and 75% quantiles. *Colored areas* 95% confidence intervals (*CI*)

Median OS was 22.1, 20.9, and 13.1 months for patients with high, intermediate, and low GTV2, respectively, before the initiation of boost RT, Fig. 2. The HR for patients with intermediate (35.7–130.8 ml) and high GTV2 (133–589.5 ml) vs. low GTV2 (<35.7 ml) was 1.42 (0.89–2.26, *p* > 0.05) and 2.06 (1.16–3.64, *p* = 0.01), respectively. In the adjusted model, the HR was 1.64 (0.94–2.87, *p* > 0.05) for low vs. medium GTV2 and 2.75 (1.12–6.75, *p* = 0.03) for low vs. high GTV2, respectively (Table 2). In the model adjusted for GTV1, the HR was 1.44 (0.89–2.34, *p* > 0.05) for low vs. intermediate GTV2 and 2.28 (0.98–5.29, *p* > 0.05) for low vs. high GTV2.

**Table 2:** Hazard ratios for different GTV parameters from Cox-regression models with the outcome of overall survival

	Crude model		GTV1 adjusted		Adjusted model^a^	
**GTV1 [per 300 mL]**						
Low: <47.13	1		–	–	1	
Medium: 47.8–217.6	1.04 (0.76–1.42)	ns	–	–	1.02 (0.71–1.44)	ns
High: 220.0–877.0	1.49 (1.04–2.14)	0.03	–	–	1.34 (0.90–2.00)	ns
**GTV2 [per 300 mL]**						
Low ≤35.71	1				1^b^	
Medium: 35.72–130.8	1.42 (0.89–2.26)	ns	1.44 (0.89–2.34)	ns	1.64 (0.94–2.87)^b^	ns
High: 133.0–589.5	2.06 (1.16–3.64)	0.01	2.28 (0.98–5.29)	ns	2.75 (1.12–6.75)^b^	0.03
**Relative Decrease [per 50%]**						
Low <12.7%	1				1	
Medium: 13.1–48.1%	0.92 (0.53–1.58)	ns			0.7 (0.38–1.27)	Ns
High: 48.3–79.8%	1.01 (0.64–1.59)	ns			0.8 (0.48–1.33)	Ns

^a^Adjusted for T-stage, chemotherapy, age, RT-dose, histology (Adeno or squamosa), grading, pulmonary comorbidities

^b^addtitionally adjusted for GTV1

**Fig. 3:**
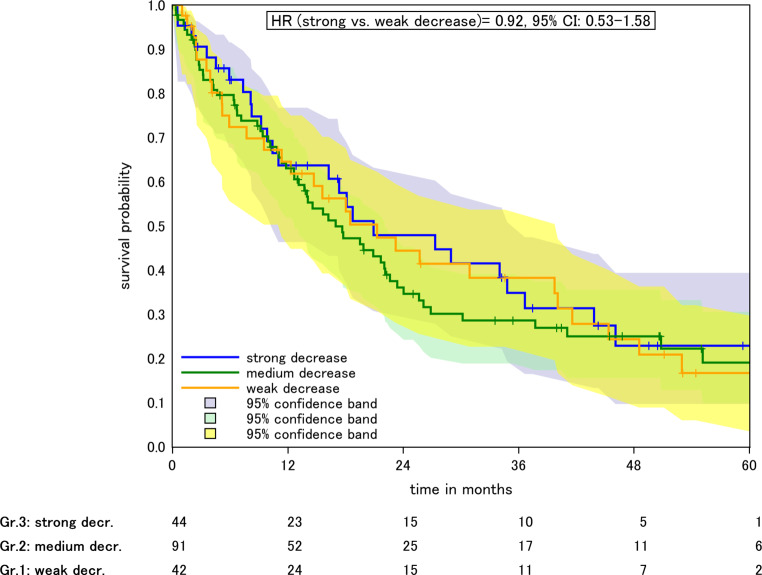
Kaplan–Meier plot of OS according to relative GTV change during radiotherapy (from GTV1 to GTV2). Weak, medium, and strong GTV decrease referring to the 25 and 75% quantiles. *Colored areas* 95% confidence intervals

Patients with a strong (48.3–79.8%), intermediate (13.1–48.1%), and weak (< 12.7%) tumor volume decrease during RT (i.e., from GTV1 before RT to GTV2) had a median OS of 20.9, 17, and 21.2 months, respectively, Fig. 3. In the crude model, HR was 0.92 (0.53–1.58, *p* > 0.05) for weak vs. intermediate and 1.01 (0.64–1.59, *p* > 0.05) for weak vs. strong decrease. In the adjusted model, HR was 0.7 (0.38–1.27, *p* > 0.05) for weak vs. intermediate and 0.8 (0.48–1.33, *p* > 0.05) for weak vs. strong GTV decrease during RT (Table 2).

When only patients with available data on GTV1 and GTV2 were considered (*n* = 176), absolute GTV1 before RT was not significantly associated with OS in the unadjusted (HR 0.71, CI 0.36–1.38, *p* > 0.05, see supplement) or adjusted model (HR 0.69, CI 0.32–1.49, *p* > 0.05).

Absolute GTV2 was significantly associated with OS. Here, for an additional 300 ml in GTV2, the HR increased by 3.18 (1.07–9.45, *p* = 0.04) in the crude and by 3.7 (1.01–13.52, *p* = 0.04, see the supplement) in the adjusted model.

The absolute GTV decrease from GTV1 to GTV2 was significantly associated with OS: Every decrease of 50 ml led to a 0.8-fold risk (CI 0.64–0.99, *p* = 0.04). There was no evidence for an association of relative GTV change with OS (HR = 0.75, 95% CI 0.47–1.21, *p* > 0.05, supplement).

In the subgroup of patients with a PET scan (*n* = 264), survival analyses did not differ significantly with regard to GTV delineation based on integrated PET-CT vs. separate PET and CT.

When adenocarcinoma patients were compared with SCC patients, the HR for the absolute GTV reduction from GTV1 to GTV2 was 0.98 (95% CI 0.86–1.11) in the crude and 0.66 (0.47–0.93) in the adjusted model for SCC. For adenocarcinoma, the HR was 1.1 (95% CI 0.94–1.3) in the crude and 1.06 (0.76–1.48) in the adjusted model.

In the sensitivity analysis adjusted for N stage (Table 3), virtually no change in the respective effect estimates were found. This holds for the analysis of SCC and AC when the relative and absolute change in tumor volume was considered. Most importantly, the hazard ratio for the absolute change in SCC showed a similar estimate, with a roughly 30% lower mortality risk per 50% decrease in volume (HR = 0.69, 95% CI: 0.49–0.96).

**Table 3:** Results from the Cox regression with tumor volume change (relative and absolute) as predictors

GTV	Histology	Complete model
Absolute	Adeno	1.06 (0.75–1.49)
	SCC	0.69 (0.49–0.96)
Relative	Adeno	2 (0.75–5.28)
	SCC	0.62 (0.34–1.13)

Models were adjusted for N stage, chemotherapy, age, RT-dose, histology (AC or SCC), grading, pulmonary comorbidities

*AC *adenocarcinoma, *SCC *squamous cell carcinoma

In the sensitivity analysis considering only cases with conventional fractionation, virtually the same effect estimates were found as in the previous analysis which included all fractionation schemes (HR = 0.81, 95% CI: 0.65–1.02).

## Discussion

To the authors knowledge, the present study which incorporates a total of 347 patients across 21 European institutions with inoperable stage III NSCLC treated by definitive CRT is one of the largest multicenter retrospective evaluations of the prognostic impact of pre-treatment GTV, GTV during RT, and its changes on outcome after RT.

Currently available studies often include only a small number of patients with partly overlapping patient cohorts. Data quality is further limited due to the highly heterogeneous GTV detection timepoints as well as the definition and detection methodology of tumor volumes. Also, most available studies include patients whose GTV was determined after (neoadjuvant) chemotherapy. In addition, three studies even combine the tumor volume of the primary tumor with affected lymph nodes [7, 10, 12]. In our study, only patients without previous chemo- or surgical therapy who were treated with definitive radio(chemo)therapy in curative intent where evaluated and the GTV was defined as gross tumor volume excluding lymph nodes.

Our data show that a high pre-RT GTV is associated with inferior overall survival. This stands in line with large available datasets evaluating pre-treatment GTV and its impact on outcome after RT such as the works from Martel, Kim, and Bradley et al., who reported a strong influence of baseline GTV before RT on OS, cause-specific survival, and tumor control [4]. However, after adjusting for age, T stage, N stage, and grading, in our study, there was no evidence for an impact of baseline GTV on OS anymore. Interestingly, these findings are consistent with results of the largest available study done by Ball et al., who (after adjusting for T and N stage) also did not find a significant association between baseline GTV and survival after radical RT [11].

Kanzaki et al. reported a significant impact of pre-treatment GTV on OS after RT for a continuous increase of 10 cm^3^ [15]. Our own data indicate a detrimental effect of GTV2 on survival for a continuous increase of 300 ml. However, the same study of Kanzaki et al. only found baseline GTV before RT to be an independent prognostic factor in patients with adenocarcinoma [15], which is suggestive of a potentially important role of histology in the GTV-guided survival prediction. The fact that the association of histology and GTV has so far not been widely evaluated further underlines the possible underestimation of the impact of histology. After adjusting for different parameters including histology, our statistical analysis did not reveal the histologic subtype to be a significant confounder. However, when patients were analyzed separately according to their histology (adenocarcinoma vs. SCC), our data showed that the survival effect of the absolute GTV reduction from GTV1 to GTV2 was more pronounced in SCC NSCLC patients compared to adenocarcinoma patients.

While the summarized evidence overall favors a prognostic quality of baseline GTV before RT, the predictive value of GTV detected during RT is much less clear [4]. The number of studies investigating the prognostic impact of intra-therapeutic GTV and the number of evaluated patients within those studies is very limited. Overall, no final agreements can be found in the literature concerning volume changes during therapy. Nonetheless, all studies report a volume reduction at the end of therapy, although the difference was not always statistically significant. In a study containing 10 patients treated with helical tomotherapy, the authors observed a relative median tumor reduction during therapy of 1.2% per day (0.6–2.3%) [16]. While patient numbers in available studies focusing on the prognostic impact of intra-therapeutic GTV range from 10–157, the present study with its 339 patients (with available GTV measurements during RT) is the largest retrospective evaluation of intra-therapeutic GTV in a homogeneous patient cohort of UICC stage III NSCLC patients undergoing definitive CRT.

In our study, GTV during RT was re-evaluated between 40 and 50 Gy. In the literature, GTV measurement timepoints vary between 2 weeks after the start of RT and 4 weeks after RT [4]. Given that response to irradiation and, consequently, tumor volume changes are relatively slow, GTV evaluation after treatment may be misleading. Thus, re-evaluation of GTV during RT was predominantly carried out between 40 and 50 Gy in order to adapt the treatment plan for tumor volume changes. Clinical evidence also suggests significant tumor volume changes after 30–50 Gy [17, 18]. While no significant effect of relative GTV changes during RT on OS was found in our dataset, absolute GTV detected during RT between 40 and 50 Gy in our study significantly impacted OS in both the crude and the adjusted model. Thus, it is the absolute volume rather than the proportion of the initial volume that predicts survival, which make these findings most relevant to patients with a high pre-treatment tumor volume.

For every continuous GTV increase during RT (300 ml), OS was significantly reduced and a continuous GTV decrease per 50 ml during RT had a positive effect on OS. In the literature, the prediction of outcome based on GTV changes during RT remains inconclusive [4]. While some studies [15, 18, 19] reported a tumor volume reduction or the reduction ratio to be associated with improved OS or PFS after RT, others (Van Elmpt et al.) could not confirm these results. There are also conflicting results indicating an inferior OS in patients with a higher tumor volume reduction during RT [20]. Thus, the predictive value of GTV changes during RT remains controversial [4].

When interpreting the results of this study, certain limitations must be considered. In the literature, mostly small patients case numbers and considerable variations in GTV measurement methods, GTV readout timepoints, and treatment regimens significantly limit the informative value of published studies investigating the prognostic value of GTV before and during RCT of advanced NSCLC. In addition, varying and incongruent GTV definitions as well as frequently inadequate staging further complicate comparability between trials [4]. The large number of stage III NSCLC patients (*n* = 347) clearly exceeds the patient number of most of the published studies. However, despite the fact that the inclusion and patient selection criteria in this study were well defined, selection effects and bias at the individual-center level cannot be fully excluded.

In addition, even though the patient cohort in this study was relatively homogeneous regarding treatment, with an absolute majority of 87% of patients having received a total dose of 60 Gy and conventional fractionation in 91% with concurrent CRT in 72% of patient cases, a certain level of heterogeneity regarding radiation dose, fractionation, and CT timing needs to be acknowledged by the authors.

Furthermore, the retrospective character of this study with all its inherent limitations, as opposed to a purely prospective study, needs to be taken into account.

Our study focused on a cohort of UICC stage III A and B NSCLC patients in equal distribution. Nevertheless, a certain variability cannot be excluded with regard to inter-center inconsistencies, treatment regimen variability, and different imaging methods for GTV re-evaluation. However, a statistically significant difference in GTV delineation based on PET-CT vs. separate PET and CT could not be determined. The GTV definition and 3D radiotherapy planning in this study was uniform and imaging was identically performed at centers for GTV1 and GTV2 detection. However, (intra-therapeutic) GTV2 was not available for all patients and potential variations between centers in PET-CT co-registration methods need to be considered.

In our study, iv contrast was used for CT/PET-CT to support GTV contouring, but as GTV in this study was defined as the gross tumor volume excluding lymph nodes, a certain level of uncertainty and difficulty in GTV delineation and separation from adjacent lymph nodes cannot be fully excluded, particularly in stage III NSCLC patients.

In our study, it appears that the association of GTV decrease with OS was driven by the squamous cell cancer subgroup, which is known to respond well to RT compared to adenocarcinoma, where a lack of response would be associated with an increased HR for death. The lack of effect in the adenocarcinomas is thus not unexpected and needs to be taken into consideration regarding the conclusions drawn in this report. These findings are supported by the data of Kwint et al., who also reported a difference in the association of GTV changes and OS depending on tumor histology [21].

In the same study, which investigated the prognostic value of volumetric changes of the primary tumor using cone beam CTs during concurrent chemoradiation in NSCLC patients, a similar approach was reported in that patient subgroups were based on the extent of GTV volume reduction during treatment. In contrast to our results, which showed an association between GTV changes during RT and OS, Kwint et al. could not determine a significant relation between tumor volume changes and outcomes such as OS and PFS. In agreement with our own data, however, is the strong predictive quality of pre-treatment GTV.

Finally, our retrospective analysis was undertaken before the approval of adjuvant PD-L1 immunotherapy with durvalumab in stage III NSCLC after chemoradiotherapy [22, 23]. Therefore, no conclusions can be made on the interaction of tumor volume and its changes during radiotherapy with adjuvant immunotherapy.

## Conclusion

In summary, our data suggest that the prognostic quality of GTV measured during RT is more robust than the prognostic value of baseline GTV detected before RT. Furthermore, our results support the notion that absolute rather than relative GTV changes (i.e., decrease) predict a favorable prognosis. Certainly, the prognostic impact of GTV and its changes during RT remain inconclusive and large prospective clinical trials are needed to finally clarify the prognostic and, in addition, the predictive value of the tumor volume and its changes during radical RT of locally advanced NSCLC.

## Supplementary Information

Plot of the martingale residuals from multivariate Cox models in relation to the linear predictor

Hazard ra os for different GTV parameters from Cox-regression models with the outcome of overall survival.

## Abbreviations

AC: Adenocarcinoma
CHT: Chemotherapy
CI: Confidence interval
CRF: Case report form
CRT: Chemoradiotherapy
CT: Computed tomography
DKFZ: German Cancer Research Center
DKTK: German Cancer Consortium
GTV: Gross tumor volume
HR: Hazard ratio
IV: Intravenous
NSCLC: Non-small-cell lung cancer
OS: Overall survival
PET: Positron-emission tomography
PFS: Progression-free survival
RT: Radiotherapy
SCC: Squamous cell carcinoma
SD: Standard deviation
SIB: Simultaneous integrated boost
TNM: Tumor node metastasis
UICC: Union for International Cancer Control
