# Correction to: Prognostic impact of gross tumor volume during radical radiochemotherapy of locally advanced non-small cell lung cancer—results from the NCT03055715 multicenter cohort study of the Young DEGRO Trial Group

**DOI:** 10.1007/s00066-021-01750-z

**Published:** 2021-03-05

**Authors:** C. Ostheimer, M. Mäurer, N. Ebert, D. Schmitt, D. Krug, R. Baumann, C. Henkenberens, F. A. Giordano, L. Sautter, Guerra López, D. F. Fleischmann, M. Niyazi, L. Käsmann, D. Kaul, A. H. Thieme, C. Billiet, S. Dobiasch, C. R. Arnold, M. Oertel, J. Haussmann, T. Gauer, Y. Goy, C. Suess, S. Ziegler, C. M. Panje, C. Baues, M. Trommer, T. Skripcak, D. Medenwald

**Affiliations:** 1 Department of Radiation Oncology, Faculty of Medicine, Martin Luther University Halle-Wittenberg, Ernst-Grube-Straße 40, 06110 Halle (Saale), Germany; 2 Department of Radiation Oncology, University Medical Center Jena, Jena, Germany; 3 Department of Radiation Oncology, University Medical Center Dresden, Dresden, Germany; 4 OncoRay—National Center for Radiation Research in Oncology, Dresden, Germany; 5 Department of Radiation Oncology, University Hospital Heidelberg, Heidelberg, Germany; 6 National Center for Radiation Research in Oncology (NCRO), Heidelberg, Germany; 7 Heidelberg Institute for Radiation Oncology (HIRO), Heidelberg, Germany; 8 Department of Radiation Oncology, University Medical Center Schleswig-Holstein, Kiel, Germany; 9 Department of Radiation and Special Oncology, Hannover Medical School, Hannover, Germany; 10 Department of Radiation Oncology, University Medical Center Mannheim, Mannheim, Germany; 11 Department of Radiation Oncology, Hospital Universitario Virgen del Rocío, Sevilla, Spain; 12 Department of Radiation Oncology, LMU Munich, Munich, Germany; 13 partner site Munich, German Cancer Consortium (DKTK), Munich, Germany; 14 German Cancer Research Center (DKFZ), Heidelberg, Germany; 15 Department of Radiation Oncology, University of Lübeck, Lübeck, Germany; 16 Department of Radiation Oncology, Charité School of Medicine, Berlin, Germany; 17 Campus Virchow-Klinikum, University Hospital, Berlin, Germany; 18 Department of Radiation Oncology, Iridium Kankernetwerk, Antwerp, Belgium; 19 Department of Radiation Oncology, Technische Universität München, Munich, Germany; 20 Department of Therapeutic Radiology and Oncology, Medical University of Innsbruck, Innsbruck, Austria; 21 Department of Radiation Oncology, University Medical Center Muenster, Muenster, Germany; 22 Department of Radiation Oncology, University Medical Center Düsseldorf, Dusseldorf, Germany; 23 Department of Radiotherapy and Radio-Oncology, University Medical Center Hamburg-Eppendorf, Hamburg, Germany; 24 Department of Radiation Oncology, University Medical Center Regensburg, Regensburg, Germany; 25 Department of Radiation Oncology, University Medical Center Erlangen, Erlangen, Germany; 26 Department of Radiation Oncology, Kantonsspital St. Gallen, St. Gallen, Switzerland; 27 Department of Radiation Oncology and Cyberknife Center, University of Cologne, Cologne, Germany; 28 German Cancer Consortium (DKTK), Dresden, Germany

**Erratum to:**

**Strahlenther Onkol 2020**

10.1007/s00066-020-01727-4

The original version of this article unfortunately contained a mistake. The spelling of M. Trommer’s name was incorrect. The corrected author list is given above.

The original article has been corrected.

