# Supplementary material for: Prognostic impact of gross tumor volume during radical radiochemotherapy of locally advanced non-small cell lung cancer—results from the NCT03055715 multicenter cohort study of the Young DEGRO Trial Group
Source: Strahlenther Onkol. 2021 Jan 7;197(5):385–95. doi: 10.1007/s00066-020-01727-4 (PMC8062351; doi:10.1007/s00066-020-01727-4)
Supplement: Supplementary file 1 — Plot of the martingale residuals from multivariate Cox models in relation to the linear predictor [file 66_2020_1727_MOESM1_ESM.docx]

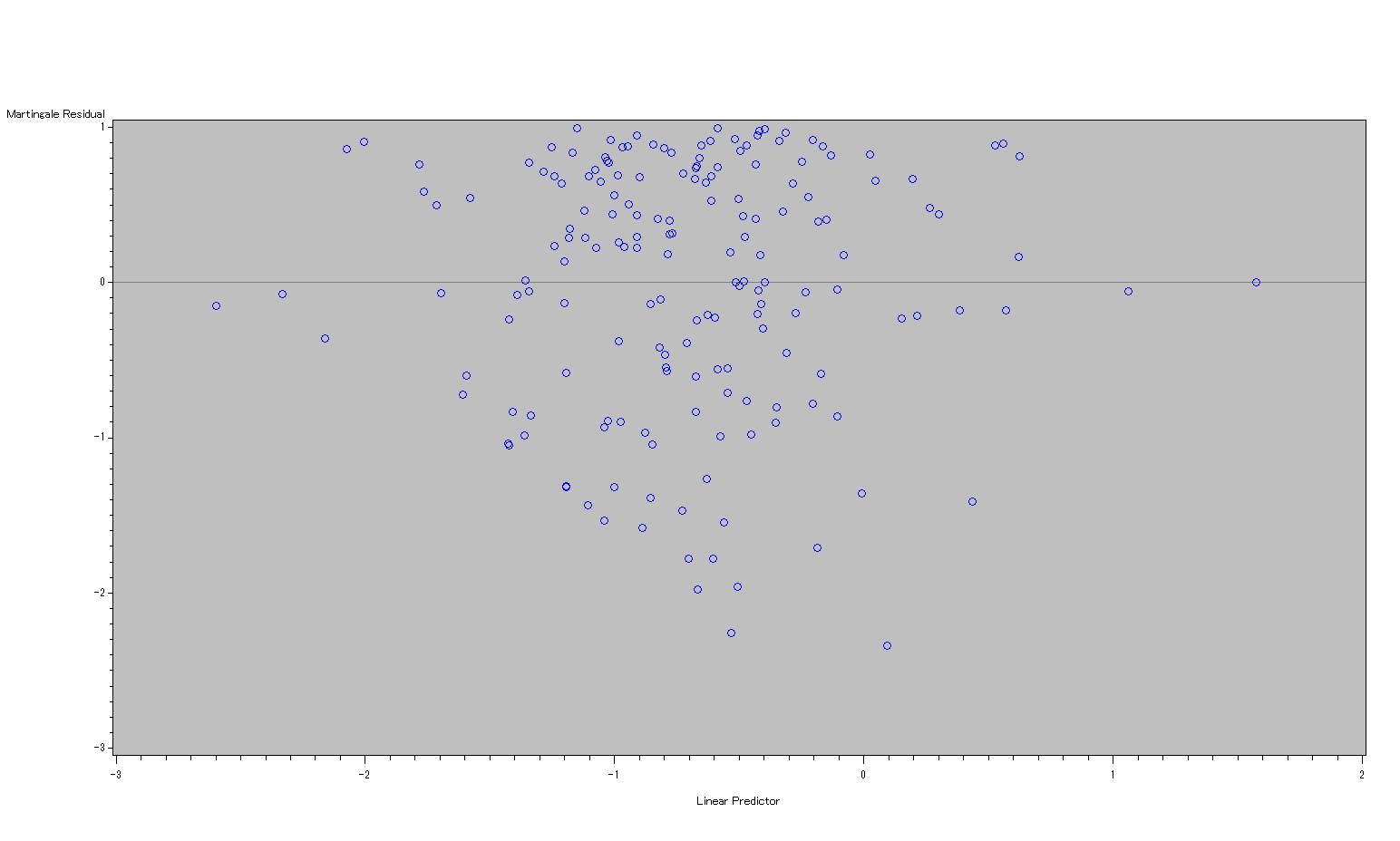


**Plot of the Martingale Residuals from Multivariate Cox Models in relation to the linear predictor**
