# Supplementary material for: Prognostic impact of gross tumor volume during radical radiochemotherapy of locally advanced non-small cell lung cancer—results from the NCT03055715 multicenter cohort study of the Young DEGRO Trial Group
Source: Strahlenther Onkol. 2021 Jan 7;197(5):385–95. doi: 10.1007/s00066-020-01727-4 (PMC8062351; doi:10.1007/s00066-020-01727-4)
Supplement: Supplementary file 2 — Hazard ra os for different GTV parameters from Cox-regression models with the outcome of overall survival. [file 66_2020_1727_MOESM2_ESM.pdf]

Both parameters

N=176	Crude model		Adjusted model		Adjusted model 2*	
GTV1 per 300mL	0.71 (0.36- 1.38)	ns	0.69 (0.32- 1.49)	ns	0.71 (0.33- 1.54)	ns
GTV2 per 300mL	3.18 (1.07- 9.45)	0.04	3.86 (1.06- 14.0)	0.04	3.7 (1.01- 13.52)	0.04

Adjusted for T-stage, chemotherapy, age, RT-dose, histology (Adeno or squamosa), grading, pulmonary comorbidities.

Model 2: Additional adjustment for PET-CT or PET + CT coregistered (Fusion)

Change: absoute or relative

N=176	Adjusted (absolute)			Adjusted (relative)	
Decrease per 50ml	0.8 (0.64- 0.99)	0.04	Decrease per 50%	0.75 (0.47- 1.21)	ns

Adjusted for T-stage, chemotherapy, age, RT-dose, histology (Adeno or squamosa), grading, pulmonary comorbidities

No change in effect estimates after adjustement for PET-CT
